# CD154 Expression Indicates T Cell Activation Following Tetanus Toxoid Vaccination of Horses

**DOI:** 10.3389/fimmu.2022.805026

**Published:** 2022-04-13

**Authors:** Christiane L. Schnabel, Babette Fletemeyer, Sabrina Lübke, Eliane Marti, Bettina Wagner, Gottfried Alber

**Affiliations:** ^1^ Institute of Immunology, Faculty of Veterinary Medicine, Leipzig University, Leipzig, Germany; ^2^ Clinical Immunology Group, Department for Clinical Research and Veterinary Public Health (VPH), Vetsuisse Faculty of the University of Bern, Bern, Switzerland; ^3^ Department of Population Medicine and Diagnostic Sciences, College of Veterinary Medicine, Cornell University, Ithaca, NY, United States

**Keywords:** TRAP, gp39, CD40L, CD40 ligand, antigen-specific, equine

## Abstract

Despite the relevance of adaptive immunity against equine pathogens antigen-specific T cell responses of horses are not well characterized and the lack of insight into T cell responses hampers the understanding of the pathogeneses of important diseases. In this study we used tetanus toxoid (TT) as a well-defined antigen to characterize antigen-reactive T cells. Six healthy adult horses received a routine booster against tetanus with an immune stimulating complex (ISCOM)-based vaccine and were followed for 28 days. TT-specific serum antibodies were quantified by ELISA and increased in all horses by day 7 after vaccination. CD154 is an established indicator of antigen-reactive T helper cells in other species, but has not been characterized in horses. CD154 detection in equine PBMC by an anti-human CD154 antibody (clone 5C8) was confirmed by Western blots and then applied for flow cytometry. As a common indicator of equine T cell activation, cytokine induction was studied in parallel. T cells were analyzed by multicolor flow cytometry of PBMC after re-stimulation with TT *in vitro*. Reactive T helper (Th) cells were characterized by increased frequencies of CD4^+^CD154^+^ lymphocytes in *in vitro* TT-re-stimulated PBMC on day 14 after vaccination of the horses compared to pre-vaccination. The majority of all CD154^+^ cells after TT re-stimulation were CD4^+^ Th cells, but CD154 was also induced on CD4^-^ cells albeit in lower frequencies. CD154^+^CD4^+^ Th cells were enriched in cytokine-expressing cells compared to CD154^-^CD4^+^ Th cells. Similar to the CD4^+^CD154^+^ frequencies, CD4^+^IL-4^+^, CD4^+^IFN-γ^+^ and CD4^+^TNF-α^+^ were increased after vaccination, but IL-4^+^ increased later than IFN-γ^+^ and CD4^+^TNF-α^+^, which already exceeded pre-vaccination frequencies on day 7. CD4^+^CD154^+^ frequencies correlated positively with those of CD4^+^IL-4^+^ (Th2) on day 14, and negatively with CD4^+^IFN-γ^+^ induction on day 7, but did not correlate with CD4^+^TNF-α^+^ frequencies or TT-specific antibody concentrations. CD154 appears to be a useful marker of antigen-reactive equine Th cells in combination with cytokine expression. The T cell analyses established here with TT can be applied to other antigens relevant for infections or allergies of horses and in horse models for translational research.

## Introduction

The horse is an important veterinary species and is gaining attention as a large animal model, for example for allergies, asthma, and regenerative medicine approaches (1–4). Particularly, in contexts of immune-mediated disorders and protection from infectious diseases the analysis of adaptive immune responses is critical. Antigen-specific serum antibodies are established markers of equine adaptive immune responses (1, 5–7). In comparison, specific T cells are more challenging to assess (8) and less characterized in horses.

Most analyses of equine T cell responses used proliferation, cytotoxicity or cytokine detection in PBMC after antigen re-stimulation *in vitro* as indicators of specific T cell reactivity (3, 5, 7, 9–12). However, analyses of tetanus-reactive T cells in PBMC yielded controversial results using proliferation assays (13, 14). Nevertheless, low frequencies of antigen-specific T cells in peripheral blood are a limitation of these assays regarding sensitivity (8) and additional markers of antigen-reactive T cells can be beneficial in the detailed analysis. Circulating antigen-reactive Th cells are mainly effector memory cells that are antigen-experienced. If their specific peptide is presented to them *via* MHC class II they are (re-)activated through their specific T cell receptor with low requirement of co-stimulation due to previous activation and strong reactivity. This reactivity to the matching antigen stimulus is indicated by induction of cytokines and surface molecules like CD154 (CD40L) after short re-stimulation (8, 15, 16).

In mice and man, CD154 has been established as a marker of T helper cell (Th) activation by antigens (8, 16, 17). CD154 is a member of the type II transmembrane protein of the TNF superfamily (17–19) with a predicted MW of 28.7 kDa in horses [GenBank accession: AQM73588.1 (20)]. In humans and mice, the predicted MW of CD154 is 29 kDa, but the actual MW of CD154 is usually 33 or 39 kDa due to post-translational modifications of the protein (17, 19, 21). CD154 can also be cleaved off its transmembrane part and released as a soluble molecule estimated to be a 18 kDa protein with cytokine-like function (21, 22). CD154 is transiently expressed after specific stimulation through the T cell receptor and indicative of the capacity for T cell help *via* CD154-CD40 ligation. CD154 ligates CD40 on antigen presenting cells and this ligation is essential for enhanced antigen presentation and adaptive immune responses of B- and T cells (8, 16, 17). Human and murine CD154 is furthermore expressed by a variety of other leukocytes including myeloid cells, platelets, and even by non-leukocytes (17, 21).

Equine CD154 has been analyzed sporadically. In equine mesenchymal stem cells CD154 RNA expression was reduced after *in vitro* stimulation with TNF-α and IFN-γ for 72h (23). Recombinant equine CD154 was used as an experimental vaccine adjuvant of a West Nile Virus subunit vaccine for horses *in vivo* and improved the induction of neutralizing antibodies compared to the subunit vaccine without CD154 (24). Furthermore, CHO cell expressed recombinant (*r*) equine CD154 stimulated equine monocyte-derived macrophages to produce reactive oxygen species *in vitro* (20). In this study, an anti-human CD154 antibody was used for the confirmation of *r* equine CD154 protein expression (20). In humans and pigs CD154 has been used as an indicator of antigen-specific T cell activation and for the enrichment of reactive T cells (8, 15, 16, 25, 26), detected with another anti-human CD154 monoclonal antibody (mAb), clone 5C8 (27).

To facilitate the analyses of equine antigen-reactive T cells we tested the mAb 5C8 for specific cross-reactivity with equine CD154. We then applied it to T cell analyses and used a tetanus booster vaccination *in vivo* as a model of provoked adaptive immune responses in horses. We demonstrated the induction of CD154 as an indicator of T cell activation with tetanus toxoid and compared it with cytokines as conventional indicators of T cell activation and with the induced TT-specific serum antibodies of the horses.

## Materials and Methods

### Horses, Vaccination, and Samples

Six healthy warmblood geldings aged 16 years (median, range 12 – 18) were used (**Supplementary Table 1**). All horses had been vaccinated regularly against tetanus throughout their life. The last vaccination was median 31 months (range 15 – 40) ago before this study, conducted from November to December 2020 (**Supplementary Table 1**) following the routine prophylaxis schedule for the herd. The horses had hay and water *ad libitum*, on pasture or in a barn with paddock access.

As part of routine prophylaxis procedures, an intramuscular tetanus toxoid booster vaccination (Equilis^®^ Te, Intervet Deutschland GmbH, Unterschleissheim, Germany) was administered to each horse (day 0) together with vaccination against Equine Influenza Virus (Equilis Prequenza, Intervet Deutschland GmbH, same injection site as tetanus) and Equine Herpesvirus type 1 (BioEquin H, Albrecht GmbH, Aulendorf, Germany, separate injection site).

The horses were examined clinically and blood samples were taken from each horse by jugular venipuncture with a vacutainer system (20G canula, sodium heparin and clot activator tubes, Becton Dickinson GmbH, Heidelberg, Germany) before - (day 0) and on days 7, 14, and 28 after tetanus vaccination. The additional venipunctures for blood sampling were approved as procedures under the animal experiment permission number TVV22/20 (Landesdirektion Sachsen, Germany).

Additionally, for confirmatory Western blot experiments and detailed phenotyping, PBMC were isolated from blood from three other healthy adult horses under the animal experiment permission number TVV22/20, and from left-over blood from two horses sampled for diagnostic purposes prior to elective surgery at the Department for Horses, Faculty of Veterinary medicine, Leipzig University, Germany.

### PBMC Isolation and Stimulation

Within three hours after blood sampling, PBMC where isolated from heparinized blood by density gradient centrifugation as previously described (5, 7). Viability was determined by trypan blue staining and 1E7 live cells/ml PBMC were cultured in supplemented medium (DMEM low glucose (1 g/l), 1% (v/v) non-essential amino acids (NEA), 2 mM L-glutamine, 50 μg/ml Gentamicin, 100 U/ml penicillin, 100 μg/ml streptomycin, 50 μM 2-mercaptoethanol, all from Sigma-Aldrich; 10% heat-inactivated FCS, Lonza, Cologne, Germany) at 37°C, 5% CO_2_ in a humidified atmosphere. PBMC were rested in medium overnight, followed by stimulation for 5 hours with Brefeldin A (10 µg/ml; Sigma-Aldrich) added for the last 3 hours. PBMC were stimulated by the addition of pure tetanus toxoid (TT, 5 µg/ml; Novartis-Behring, Marburg, Germany, obtained through Prof. Christian Jassoy, Institute for Medical Microbiology and Virology, Leipzig University, Germany), the tetanus vaccine (final dilution in culture 1:50 equaled 0.8 flocculation units/ml; Equilis^®^ Te, Intervet Deutschland GmbH, see **Supplementary Figure 5**), Staphylococcal enterotoxin B (SEB; 2 µg/ml; Sigma-Aldrich), or a combination of Phorbol-12-myristat-13-acetat (PMA; 25 ng/ml; Sigma-Aldrich) and ionomycin (1 µM; Sigma-Aldrich), in the culture medium.

To produce lysates for Western blot experiments, PBMC were used *ex vivo* or stimulated immediately after isolation *in vitro* for 3.5 h in the presence of 10 µg/ml Brefeldin A and 1µM Monensin (Sigma-Aldrich) in supplemented medium or with SEB or PMA/ionomycin as described above. The cells were harvested, counted, washed in PBS, snap frozen in liquid nitrogen and cell pellets stored at -80°C.

### Western Blots of Equine PBMC Lysates With CD154 Detection

**Figure 1:**
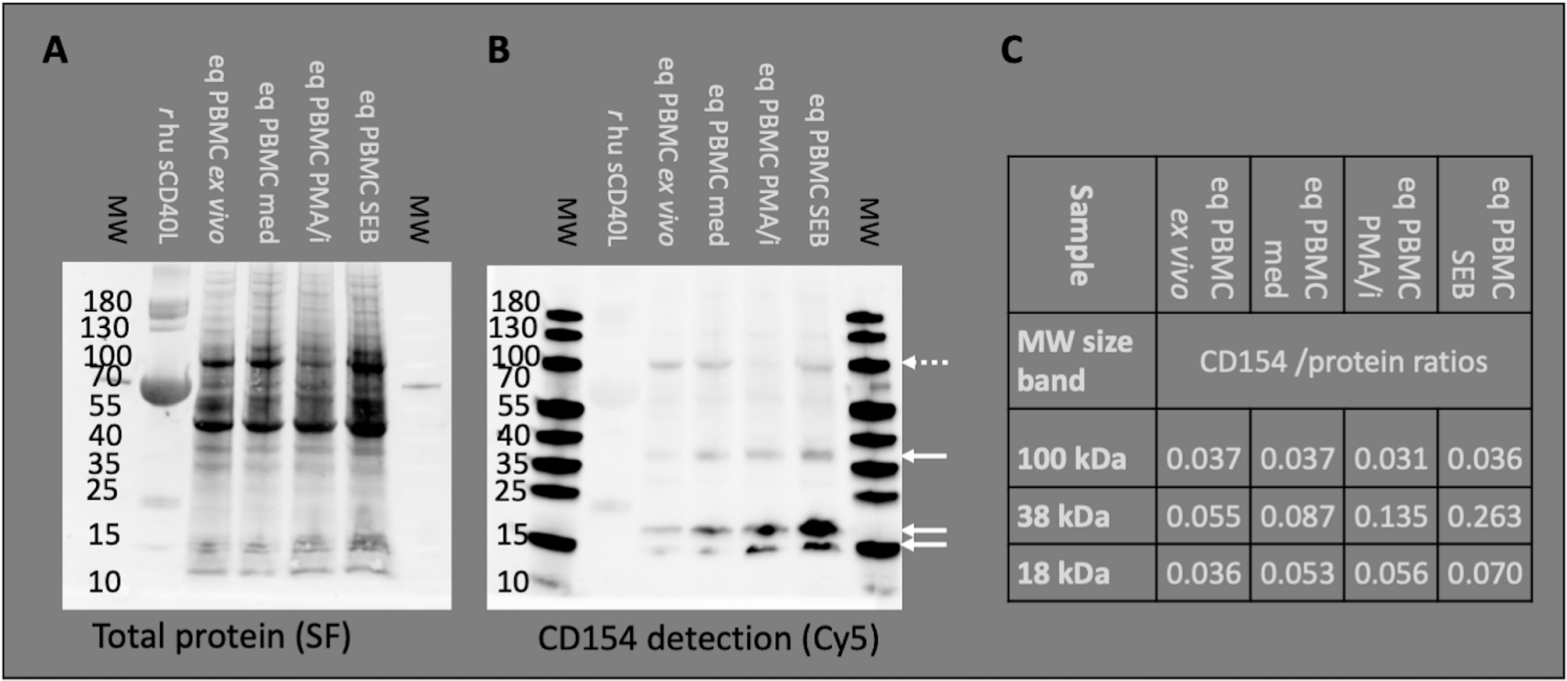
Anti-human CD154 clone 5C8 detects equine CD154 in PBMC. Representative example of Western blot experiments: Recombinant human soluble CD154 (*r* hu sCD40L) and equine PBMC lysates were separated by SDS PAGE. Molecular weights (MW) in kDa are annotated as indicated by pre-stained MW markers. **(A)** Proteins were blotted and visualized by StainFree technology (SF). Note the weak protein band of *r* hu sCD40L (15–25 kDa) accompanied by BSA (55–70 kDa, strong protein band). **(B)** The Western blots were probed with anti-human CD154 (conjugated with AF647) and detected as Cy5 fluorescence. PBMC were used *ex vivo* or after medium incubation (med), PMA and ionomycin (PMA/i)-, or SEB-stimulation *in vitro*. Arrows indicate CD154 bands (35–40 and 15–25 kDa) detected in PBMC. Occasionally, additional bands of 100 kDa were observed (dashed arrow). **(C)** CD154 detection (Cy5 fluorescence) was normalized to the total protein signal (SF fluorescence). The normalized detection/protein ratios are given per MW area and PBMC sample.

Snap frozen PBMC were lyzed in SDS-PAGE sample buffer (pH 6.8, 0.05 M Tris, 4% (w/v) SDS, 12% (x/v) glycerol, 0.01% (w/v) Bromophenol blue, 6 mM DTT, all Sigma-Aldrich) by agitation and boiling for 5 minutes. As a positive control, recombinant human CD40L stabilized with BSA (Biolegend, San Diego, CA, USA) was boiled in the same buffer. Lysates from the different PBMC treatments (protein from 1.75 E6 live cells per lane), CD40L (0.5 µg per lane), and a MW marker (PageRuler Prestained Protein Ladder (ThermoFisher Scientific) were loaded onto Mini-PROTEAN TGX Stain-Free gels (Bio-Rad Laboratories GmbH Deutschland, Feldkirchen, Germany) and separated by SDS PAGE in Tris-tricine buffer (0.1 M Tris, 0.1 M Tricine (N-[Tris(hydroxymethyl)methyl]-glycine), 0.1% (w/v) SDS, pH 8.25 (28), components from Serva Electrophoresis GmbH, Heidelberg, Germany) using a Mini PROTEAN system (Bio-Rad). The gels were UV-activated for one minute (ChemiDoc MP, Bio-Rad) to visualize proteins as StainFree fluorescent (29, 30). Then, Western blots on nitrocellulose membranes (0.2 µM Roti NC, Roth) were prepared in a tank blot procedure (25 mM Tris, 192 mM Glycine, 20% v/v methanol, all Roth) using a Mini TransBlot system (Bio-Rad). The membranes were imaged to visualize and confirm the protein transferred (StainFree, ChemiDoc MP, Bio-Rad), blocked with Serva Blue block PF (Serva) for 1h at rt and incubated with anti-CD154 conjugated to AlexaFluor647 (pure mAb from Hoelzel, conjugated with Alexa Fluor™ 647 NHS Ester, Thermo Fisher Scientific) for 1 h at rt protected from light. Bound CD154 mAb was detected as fluorescence in the Cy5 channel and matched with the protein band pattern in the StainFree channel using the ChemiDoc MP instrument with ImageLab software (Bio-Rad) applying volume tools for quantification. Double bands around 18 kDa were quantified as one band (**Figures 1A–C**). CD154 detection (Cy5 fluorescence) was normalized to the total protein signal [StainFree fluorescence (30, 31)].

### Flow Cytometric Analysis After Re-Stimulation *In Vitro*

*In vitro* cultured PBMC were collected, washed, stained with fixable viability dye eFluor™506 (eBioscience™, ThermoFisher Scientific) and fixed with 2% Paraformaldehyde in PBS (Roth). After fixation, surface staining (CD4, CD8) was performed in 3% FCS (Lonza), 0.1% NaN_3_ in PBS (Roth), followed by intracellular staining (CD154, cytokines) in 0.5% Saponin, 3% FCS, 0.1% NaN_3_ in PBS with two different antibody combinations (**Table 1**) and an isotype control panel. 300,000 events per sample were recorded with a BD LSR Fortessa™ Cell Analyzer (BD Biosciences, Ashland, OR, USA) equipped with FACS Diva™ 6.2 software (BD). Data was analyzed using FlowJo™ v10.7 software (FlowJo, LLC, BD).

**Table 1:** Antibodies for flow cytometric analysis.

Panel	Surface staining	Conjugate	Intracellular staining	References
I	CD4 biotinylated^a^ clone HB16A^1^	PE Streptavidin^3^		(32)
	CD8 DyL405^b^clone CVS8^2^		CD154 DyL488^c^ clone 5C8^4^	(25, 27)
			IL-4 AF647^d^ clone 13G7^2^	(33)
II	CD4 biotinylated^a^ clone HB16A^1^	PE Streptavidin^3^		(32)
			CD154 VioBlue clone 5C8^5^	(25, 27)
			TNF-a DyL488^c^ clone 48-4^2^,	(34)
			IFN-g AF647^d^ clone 38-1^2^	(5)

Antibody and conjugate sources: ^1^ Washington State University, Pullman, WA, USA; ^2^ Dr. B. Wagner, Cornell University, Ithaca, NY, USA; ^3^ BioLegend^®^, San Diego, CA, USA; ^4^ Hoelzel, Cologne, Germany; ^5^ Miltenyi Biotec, Bergisch Gladbach, Germany; conjugated with. ^a^ EZ-Link™ Sulfo-NHS Biotin. ^b^ DyLight™ 405 NHS Ester. ^c^ DyLight™ 488 NHS Ester, or ^d^ Alexa Fluor™ 647 NHS Ester, (all Thermo Fisher Scientific, Rockford, IL, USA).

**Figure 2:**
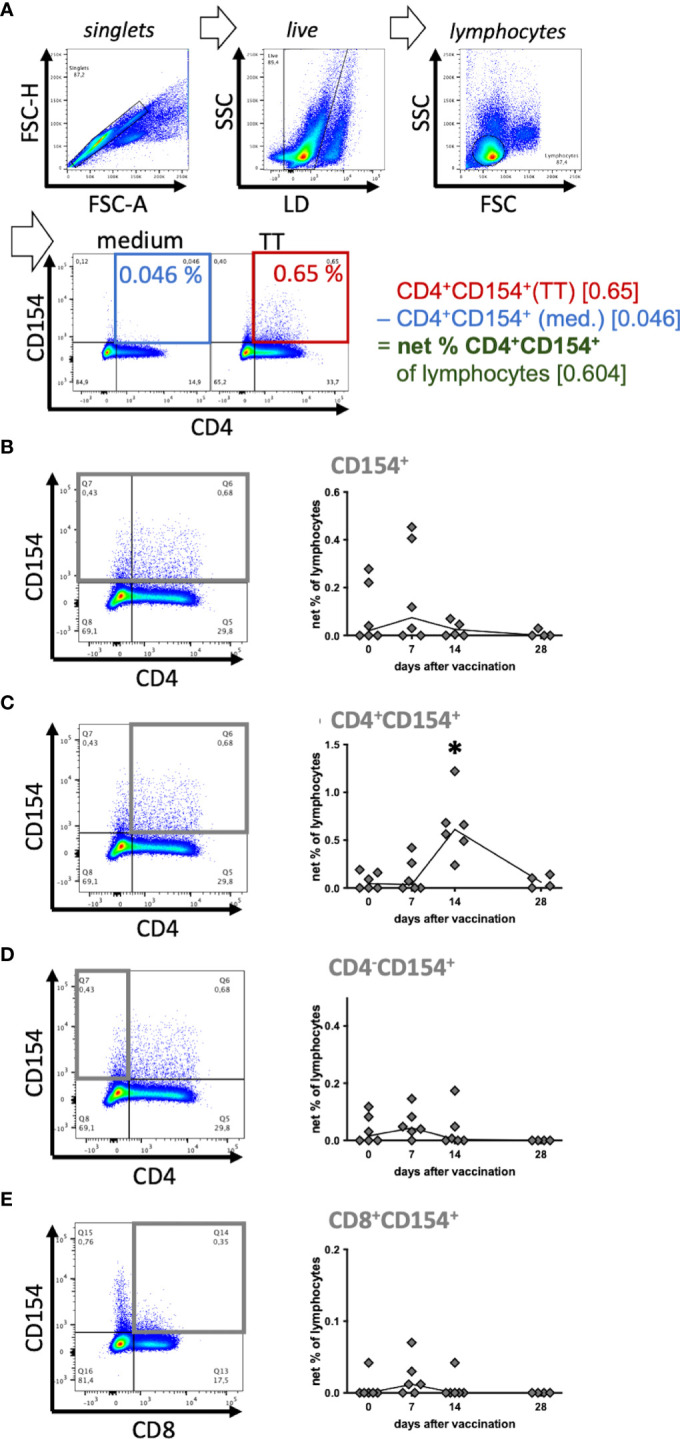
Tetanus toxoid-reactive CD154^+^ T helper cells increased after vaccination. Six horses were booster vaccinated against tetanus toxoid on day 0 and antigen-reactive lymphocytes in peripheral blood were analyzed before (0) and on days 7, 14 and 28 after vaccination. PBMC were re-stimulated with tetanus toxoid (TT) as an antigen *in vitro*, compared to medium incubation for 5h. **(A)** Pseudocolor plots of a representative sample (day 14) illustrate the gating strategy and data reduction. Doublets, and dead cells (live-dead (LD) staining) were excluded, and lymphocytes were gated by forward scatter (FSC) and side scatter (SSC) characteristics resulting in a population of (singlet live) lymphocytes. Percentages of cell populations (e. g. CD4^+^CD154^+^) were quantified in quadrant gates (% of lymphocytes). To analyze stimulation effects, baseline CD154 expression in medium (blue) was subtracted from that in stimulated (TT, red) lymphocytes resulting in (medium-corrected) net % of lymphocytes (green). **(B–E)** Net % of lymphocytes of **(B)** CD154^+^, **(C)** CD4^+^CD154^+^, **(D)** CD4^-^CD154^+^, and **(E)** CD8^+^CD154^+^ are plotted over time. The connecting line depicts medians of n=6 (day 28 n=4). Significant differences (p<0.05) after vaccination compared to pre-vaccination frequencies are indicated as * for CD4^+^CD154^+^ on day 14.

After doublet exclusion by Forward scatter (FSC) height against FSC area characteristics (singlets) live cells were selected by exclusion of viability dye positive cells. Then, lymphocytes were gated by FSC and side scatter (SSC) characteristics. The activation markers CD154 and cytokines were analyzed in comparison to isotype or fluorescence-minus-one controls in quadrant gates against CD4 as % of live lymphocytes (**Figure 2A**). All frequencies were medium-corrected by subtraction of the percentages in medium incubated samples from those after stimulation *in vitro*, resulting in net % of live lymphocytes in stimulated PBMC (**Figure 2A**). Negative values were set to 0 and induction by stimulation was analyzed (positive net %).

### Tetanus-Specific Serum Antibodies (ELISA)

Blood in clot activator tubes was allowed to clot at room temperature for six hours and was stored at 4°C overnight. The serum was then separated by centrifugation and stored at -80°C until analysis within two months after collection.

As relative standards, serum of pre-screened sera from three horses with high tetanus toxoid (TT)-binding serum antibody titers was pooled. From this serum pool immunoglobulins (Ig) were purified with Protein G as previously described (35, 36). Briefly, serum was diluted in sodium phosphate buffer and Ig allowed to bind to a HiTrap Protein G column (Cytvia, Freiburg, Germany) on an ÄKTA pure FPLC instrument (Cytvia). After washing with sodium phosphate buffer, the first elution was performed with 0.1 M glycine at pH 3 to elute primarily IgG1, followed by a second elution with 0.1 M glycine at pH 2 to elute the remaining bound IgG isotypes including IgG4/7. The eluted purified IgG fractions were neutralized with Tris (Sigma-Aldrich, Merck KGaA, Darmstadt Germany), dialyzed (SpectraPor dialysis tubing 12-14kD MWCO, VWR International, Darmstadt, Germany) against excess PBS, and concentrated with VivaSpin concentrator columns (Sigma-Aldrich). Their total protein concentration was determined with the Bradford method (Roti^®^Quant, Roth, Karlsruhe, Germany). The purified IgG eluates were used as a relative standard on ELISAs to quantify TT-specific Ig as µg/ml Ig equivalents (total Ig concentration, **Supplementary Figure 1A**).

ELISA plates (Nunc Maxisorp flat bottom plates, ThermoFisher Scientific) were coated with 1 µg/ml *Clostridium tetani* tetanus toxoid (# 1002, Hoelzel) in 50 mM sodium carbonate buffer overnight at 4°C, blocked afterwards with PBS containing 0.5% BSA and 0.1% gelatin (all reagents from Roth) for 1h at room temperature (rt), and washed three times with PBST (PBS, 0.5% Tween20, Roth). Blanks (PBST), standards (2-fold serial dilutions of pool serum and purified IgG eluates, **Table 2**), low titer control sera, and test sera were diluted in PBST and incubated for 2 h, at rt, followed by washing in PBST, incubation with biotinylated detection antibodies (**Table 2**), washing in PBST, incubation with Peroxidase-conjugated streptavidin (Jackson ImmunoResearch, Dianova, Hamburg, Germany), and washing again in PBST. 3,3’,5,5’-Tetramethylbenzidine substrate (medac GmbH, Wedel, Germany) was added for 10 min, the reaction was stopped with phosphoric acid and optical densities (ODs) quantified at 450 nm on a SpectraMax 340 reader with SoftMax Pro software (Molecular Devices, ThermoFisher Scientific). Blank-corrected ODs were used to create hyperbola standard curves and deduct concentrations (µg/ml Ig equivalents, **Supplementary Figure 1B**) with GraphPadPrism 7 (GraphPad software inc., La Jolla, CA, USA).

**Table 2:** Tetanus toxoid-specific serum immunoglobulin ELISA specifications.

Ig/isotype	Pan-Ig	IgG1	IgG3/5	IgG4/7
Detection antibody	Biotin-SP AffiniPure Goat Anti-Horse IgG (H+L)	Anti-IgG1 CVS45 biotinylated*	Anti-IgG3/5 586 biotinylated*	Anti-IgG4/7 CVS39 biotinylated*
Reference	JacksonImmuno Research 108-065-003	(37, 38)	(38)	(37, 38)
Purified Ig standard, Maximum Ig concentration	pH 2 eluate, 5 µg/ml	pH 3 eluate, 10 µg/ml	pH 2 eluate, 10 µg/ml	pH 2 eluate, 5 µg/ml
Test sera dilution	1:4000	1:500	1:4000	1:1000

*monoclonal antibodies were biotinylated with EZ-Link™ Sulfo-NHS-Biotin (ThermoFisher Scientific) as previously described (31, 38).

### Statistical Analysis

Statistical analyses were performed with GraphPad Prism software version 9.3.1 (GraphPad Software). P-values below 0.05 were considered significant for all analyses. Normal distribution of the data was tested and confirmed by Shapiro-Wilk tests. Ig concentrations and net % lymphocytes were compared after vaccination to those before vaccination with Fisher’s LSD tests after 2-way repeated measures ANOVA. Correlations between different net % of lymphocytes, Ig concentrations, or their increases (Δd0) were analyzed by Spearman correlation.

## Results

### CD154 Is Specifically Detected in Stimulated Equine PBMC

Our interest was to ascertain detection of reactive T cells in horses using tetanus toxoid as a model antigen. We aimed to analyze CD154 expression as a new indicator of equine T cell activation and validated the mAb 5C8 for detection of equine CD154.

Binding of equine CD154 (CD40L) by the anti-human CD154 mAb (5C8) was tested on Western blots in equine PBMC lysates (**Figures 1A–C**). The anti-human CD154 mAb detected equine CD154 as bands of 38 (35–40, putative membrane-bound CD154) and 18 (15–25, putative soluble CD154) kDa on Western blots of equine PBMC lysates (**Figure 1B**). Occasionally, additional bands around 100 kDa were also detected (example in **Figure 1B**). CD154 protein was detected in lysates of PBMC *ex vivo*, after medium incubation, and PMA/ionomycin, or SEB stimulation *in vitro*. The ratio of CD154 to total protein increased after stimulation with PMA/ionomycin, or SEB compared to *ex vivo* or medium incubation in the 38 kDa band. Stimulation only mildly increased CD154 detection in the 18 kDa bands, and did not affect the 100 kDa band (**Figure 1C**).

Aiming to use CD154 as a specific marker for CD4^+^ Th cell activation we also analyzed lysates of whole PBMC compared to CD4-enriched and CD4-depleted cells after *in vitro* stimulation with PMA/ionomycin. Equine CD154 was detected in both fractions with similar intensity (**Supplementary Figure 2**).

According to the consistent detection of bands of 38 and 18 kDa matching the predicted MW of membrane-bound and cleaved (soluble) equine CD154, respectively (20, 22), the anti-human CD154 mAb 5C8 was considered specifically cross-reactive with equine CD154 and used in the following flow cytometry experiments.

### Circulating TT-Reactive CD154^+^ T Helper Cells Increase After Vaccination

CD154 was analyzed by flow cytometry after *in vitro* stimulation of PBMC, fixation and intracellular antibody staining in singlet live lymphocytes. CD154 was detected in CD4^+^ Th cells and CD4^-^ cells (**Figure 2A**) matching the Western blot results. As a positive control stimulus SEB induced CD154 in all equine PBMC at all sampling time points (compare **Supplementary Figures 3**–**5**). Furthermore, *in vitro* stimulation of PBMC from vaccinated horses with TT induced increased CD154 expression in PBMC compared to medium incubation (**Figure 2A**).

Equine T cell responses against TT indicated by CD154 expression after TT re-stimulation were analyzed over time (**Figures 2B–E**) and increased net frequencies of CD154^+^ Th cells were detected after vaccination. The net % of CD4^+^CD154^+^ were clearly increased on day 14 after vaccination (median 0.61%) compared to pre-vaccination (median 0.045%; p<0.0001, **Figure 2C**). Their median net % and increase exceeded those of the other fractions analyzed. On day 7 after vaccination the median net % of CD154^+^ lymphocytes (0.075%; **Figure 2B**) and the other CD154^+^ subpopulations (0.045% CD4^-^CD154^+^, 0.012% CD8^+^CD154^+^; **Figures 2C–E**) were mildly elevated compared to pre-vaccination (0 – 0.02%, not significant, ns), but all net % returned to baseline thereafter, except for those of CD4^+^ Th cells. Net % of CD4^-^CD154^+^ and CD154^+^ live lymphocytes were lower than those of CD4^+^CD154^+^ because medium incubated CD4^-^ lymphocytes already expressed CD154 and thus reduced the net % after medium-correction (compare **Figure 2A**).

The CD154^+^ lymphocytes after medium incubation were mainly CD4^-^CD8^-^. In additional experiments we demonstrated that these cells included B cells, monocytes and other cells within PBMC, which could not be identified by the markers available (**Supplementary Figures 3**, **4**). The largest differences of CD154 expression between stimulated and medium incubated lymphocytes was consistently detected in CD4^+^ Th cells (**Supplementary Figures 3B**, **4B**) resulting in the greatest net increase after vaccination compared to the other subsets (**Figure 2C**).

In summary, CD154 expression indicated activation of CD4^+^ Th cells in response to re-stimulation *in vitro* and these reactive Th cells increased in peripheral blood after vaccination of the horses. CD154 may accordingly be used as a new indicator of reactive T cells in horses.

### CD154^+^ T Helper Cells Are Enriched in Cytokine Expressing Cells Confirming Activation in Response to Tetanus Toxoid

**Figure 3:**
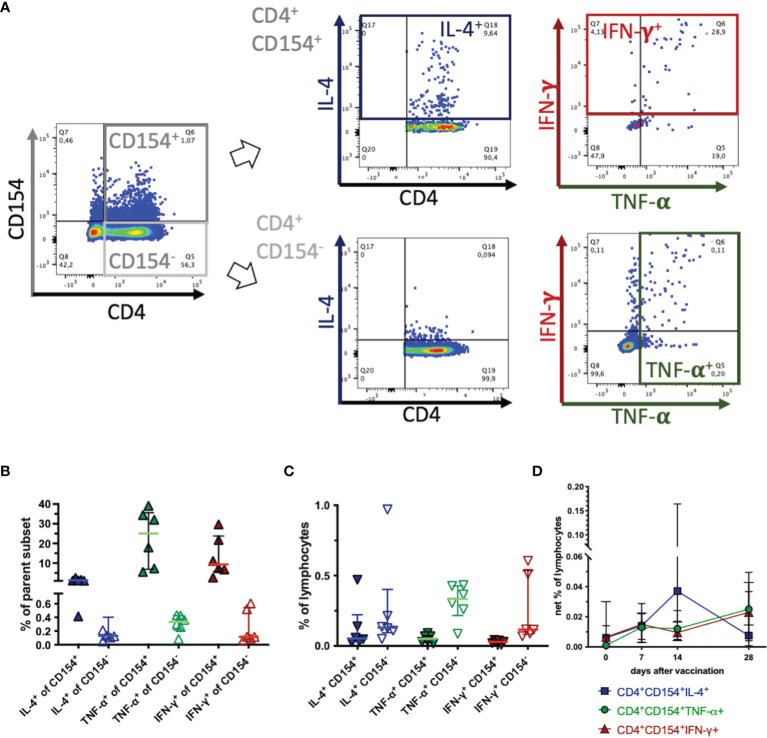
CD154 marks activated, cytokine expressing T helper cells. PBMC were re-stimulated with tetanus toxoid *in vitro* and analyzed by flow cytometry. Pseudocolor plots with enlarged dots illustrate gating in one example on day 14 after vaccination. **(A)** In singlet live lymphocytes CD4^+^CD154^+^ and CD4^+^CD154^-^ were gated. These subsets were analyzed for cytokine expression of IL**-**4 (blue), TNF-α (green), and IFN-γ (red) as the sum of two quadrants each. **(B)** The percentages of each fraction (CD154^+^ filled symbols, CD154^-^ open symbols) of the parent population are plotted. **(C)** The percentage of each fraction are calculated and plotted as percentages of all live lymphocytes (without medium correction). Horizontal bars represent medians and interquartile ranges of n=6. **(D)** CD4^+^CD154^+^cytokine^+^ were analyzed and are plotted as net % of live lymphocytes (with medium correction) before (0) and on days 7, 14 and 28 after vaccination. Medians and interquartile ranges of n=6 (day 28 n=4) are depicted. Differences after vaccination compared to pre-vaccination were not statistically significant (ns).

**Figure 4:**
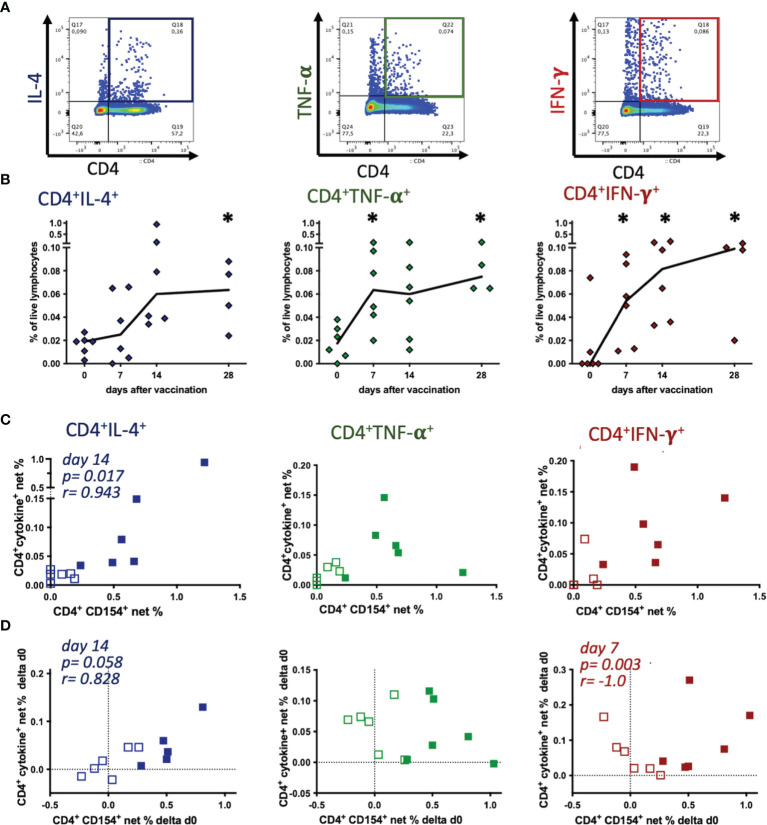
Tetanus toxoid-reactive Th cells express several cytokines, but CD154 expression mainly correlates with IL-4 induction. PBMC were re-stimulated with tetanus toxoid *in vitro* compared to medium incubation. **(A)** Pseudocolor plots with enlarged dots of a representative sample (singlet live lymphocytes) illustrate the gating of each cytokine. **(B)** Net % of live lymphocytes of CD4^+^IL-4^+^, CD4^+^TNF-α^+^, and CD4^+^IFN-γ^+^ before (0) and on days 7, 14 and 28 after vaccination are plotted. Connecting lines represent medians of n=6 (day 28 n=4). Significant differences after vaccination compared to pre-vaccination frequencies are indicated as * for the respective days (p<0.05). **(C, D)** Comparisons of net % of TT-reactive CD4^+^CD154^+^ vs. CD4^+^IL-4^+^, CD4^+^TNF-α^+^, or CD4^+^IFN-γ^+^ of lymphocytes are plotted. **(C)** Net % on day 0 (pre-vaccination, open symbols) and day 14 (filled symbols), or **(D)** the increase (Δd0) calculated as the difference between days 7 (open symbols) or 14 (filled symbols) and pre-vaccination (day 0) are plotted. Dotted lines represent 0 difference, for orientation. Spearman correlations are indicated by p and r.

Co-expression of CD154 and cytokines as indicators of cellular activation was analyzed using TT antigen stimulus *in vitro*, on day 14 after vaccination, when the highest net % of CD154^+^CD4^+^ cells (see **Figure 2C**) and net induction of the cytokines analyzed (see below, **Figure 4**) were detected. CD4^+^CD154^+^ Th cells contained higher proportions of IL-4^+^, TNF-α^+^, and IFN-γ^+^ cells than CD4^+^CD154**^-^** Th cells (**Figures 3A, B**). This was the case for CD4^+^ as well as for CD4^-^ subpopulations analyzed separately, and for all lymphocytes (data not shown). IL-4 was detected in median 7%, TNF-α in median 37%, and IFN-γ in median 26% of the CD4^+^CD154^+^ cells clearly exceeding the percentages in CD4^+^CD154**^-^** cells (**Figure 3B**). Nevertheless, CD4^+^CD154^-^cytokine^+^ lymphocytes were as well detected, particularly for TNF-α and IFN-γ. However, the CD154^-^cytokine^+^ lymphocytes were also detected after medium incubation, without additional stimuli *in vitro* and comprised more CD4^-^ than CD4^+^ cells (**Supplementary Figure 5**). Consequently, not all cytokine expressing cells were marked by CD154 and CD4^+^CD154^-^cytokine^+^ % of lymphocytes exceeded those of CD4^+^CD154^+^cytokine^+^ % in the case of TNF-α^+^, and IFN-γ^+^, and were similar between CD4^+^CD154^-^ and CD4^+^CD154^+^ in the case of IL-4 (**Figure 3C**, without medium correction). Co-expressing T helper cells (CD4^+^CD154^+^cytokine^+^) after TT-re-stimulation were analyzed over time (**Figure 3D**), but were not statistically significantly different from pre-vaccination frequencies. The net % CD4^+^CD154^+^IL-4^+^ of lymphocytes showed a similar median time course as that of CD4^+^CD154^+^ (**Figure 2C**), but the interindividual variation was high. Net % of CD4^+^CD154^+^TNF-α^+^ and CD4^+^CD154^+^IFN-γ^+^ lymphocytes increased only by day 28, but were similar after vaccination compared to day 0 (all days ns, p>0.05, **Figure 3D**).

In summary, CD154^+^ Th cells were enriched in cytokine-producing cells indicative of T cell activation, but CD154 did not mark all TT-reactive cytokine producing Th cells. Simultaneous expression (CD154^+^cytokine^+^) was detected in different proportions of the cells for the three cytokines analyzed here. Despite higher enrichment of TNF-α^+^ and IFN-γ^+^ than IL-4^+^ as % of the CD4^+^CD154^+^ cells (**Figure 3B**) the highest proportion of CD154^+^cytokine^+^ co-expression in the Th cells was observed for CD154 with IL-4 after TT restimulation (**Figure 3D**).

### Cytokine Expression After Vaccination Reveals Distinct Dynamics of Antigen-Reactive T Helper Cell Subpopulations

In order to identify all potential TT-reactive Th cells we analyzed cytokine expression over time separately as an established indicator of T cell activation and of Th cell polarization (**Figures 4A, B**) and then compared these to the dynamics of the CD4^+^CD154^+^ Th cells. After TT re-stimulation *in vitro*, net % of CD4^+^cytokine^+^ Th cells increased after vaccination (**Figure 4B**). CD4^+^IL-4^+^ Th cell net % were detectable before vaccination (median 0.019%), their median remained similar on day 7 (0.025%), and increased on day 14 (0.060%, ns) and day 28 (0.064%, p=0.049) compared to pre-vaccination (**Figure 4B**). Few CD4^+^TNF-α^+^ lymphocytes were already detectable before vaccination (day 0, median 0.018%), their median net % increased clearly by day 7 (0.064%, p=0.023), and slightly further until day 28 after vaccination (0.075%, p=0.024). TT-re-stimulated CD4^+^IFN-γ^+^ were not detected in PBMC from most horses before vaccination, but increased continuously from day 0 till day 28 after vaccination (median 0.099%) and their net % exceeded pre-vaccination frequencies all time points (p<0.05) compared to day 0 (**Figure 4B**). Comparing the three cytokines analyzed in TT re-stimulated CD4^+^ Th, IL-4 expression increased by day 14 only, and lagged behind TNF-α and IFN-γ, which were already increased on day 7 after vaccination.

TT antigen re-stimulation *in vitro* yielded clear increases of activated Th cells detected as cytokine expressing CD4^+^ cells by flow cytometry, similar to the results of the CD4^+^CD154^+^ cells. Next, we analyzed if CD154 and cytokine expression in CD4^+^ Th cells in PBMC of the individual horses were correlated as two indicators of T cell activation despite the lack of stringent co-expression of CD154 and the cytokines (**Figure 3**). On day 14 after vaccination the net % of CD4^+^CD154^+^ cells only correlated with those of CD4^+^IL-4^+^ (p=0.017, r=0.943), but not with TNF-α^+^ or IFN-γ^+^ Th cell frequencies (**Figure 4C**). Also, pre-vaccination frequencies (day 0) of CD154 and cytokine expressing CD4^+^ Th cells appeared independent (**Figure 4C**). Furthermore, increases (Δd0) of CD4^+^CD154^+^ and CD4^+^IL-4^+^ net % correlated positively on day 14 after vaccination (p=0.058, r=0.828, **Figure 4D**). In contrast, the increases of net % CD4^+^CD154^+^ did not correlate with those of CD4^+^TNF-α^+^, and correlated negatively with net % CD4^+^IFN-γ^+^ (p=0.003, r=-1.0) on day 7 after vaccination.

These findings are in agreement with the greater similarity in net % time courses after vaccination of CD4^+^CD154^+^ (**Figure 2C**) and CD4^+^IL-4^+^ compared to CD4^+^TNF-α^+^ and CD4^+^IFN-γ^+^ cells (**Figure 4B**).

### Tetanus Toxoid (TT)-Specific Serum Antibodies Are Dominated by IgG3/5, but Several Isotypes Are Boosted After Vaccination

**Figure 5:**
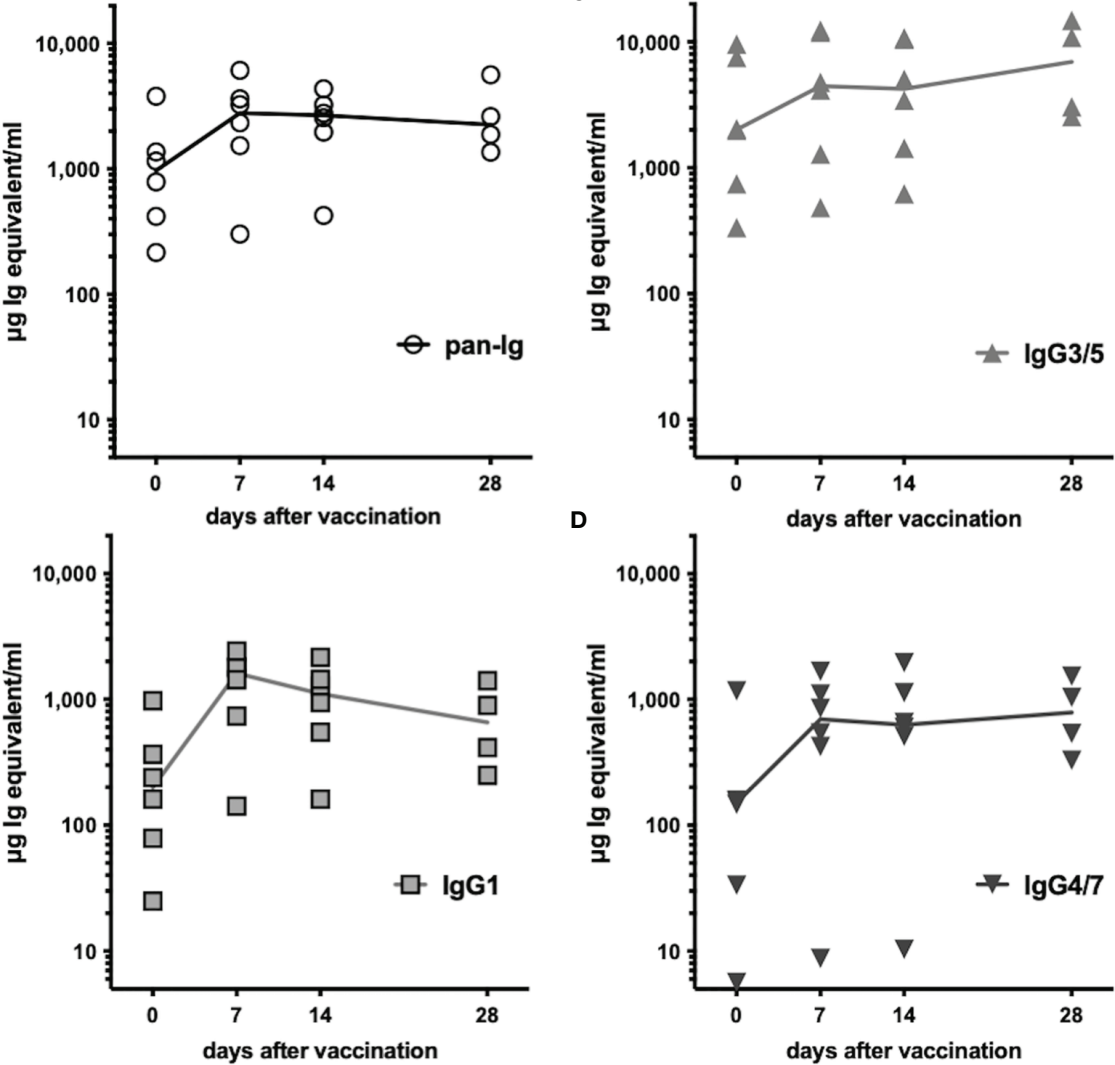
Tetanus-specific serum antibodies increased after vaccination. Tetanus toxoid-specific serum antibodies were quantified by ELISA before and after booster vaccination. Serum concentrations of **(A)** pan-Ig, **(B)** IgG1, **(C)** IgG3/5, and **(D)** IgG4/7 are plotted for each horse and time point. Connecting lines represent medians of n=6 (day 28 n=4). Compared to pre-vaccination (day 0) the concentrations of each isotype were statistically significantly increased (p<0.05, except IgG3/5 on day 28 p=0.08) at all time points after vaccination (not depicted).

All horses had previously been vaccinated with TT (**Supplementary Table 1**) and had pre-existing TT-specific serum antibodies before booster vaccination. Serum antibody concentrations additionally increased after vaccination within 7 days (**Figure 5**). Total TT-specific serum antibodies (pan-Ig) increased in all horses by day 7 after vaccination and remained elevated compared to pre-vaccination concentrations throughout the study, until day 28 (p<0.01 for all time points, **Figure 5A**). TT-specific antibody concentrations of all three isotypes analyzed were also higher at all time points after vaccination compared to pre-vaccination (p<0.05; IgG3/5 on day 28 p=0.09, **Figures 5B–D**). Pre-existing antibodies were mainly IgG3/5 isotypes. After vaccination, TT-specific IgG3/5 also had the highest concentrations of all isotypes measured, followed by IgG1 and IgG4/7. TT-specific IgG1 increased and reached a maximum on day 7 post-vaccination, but decreased thereafter still exceeding the pre-vaccination concentrations until day 28 (**Figure 5B**). IgG3/5 and IgG4/7 concentrations increased by day 7 and remained elevated until the end of the study (day 28, **Figures 5C, D**, respectively). The increases in TT-specific serum antibodies confirmed a rapidly boosted adaptive immune response to TT after vaccination of all horses.

Finally, we analyzed if TT-specific antibodies and TT-reactive Th cells in peripheral blood correlated. Net frequencies and concentrations, or their respective increases (Δd0) were mostly independent (**Supplementary Figure 6**). The only correlations observed were between CD4^+^TNF-α^+^ and pan-Ig or IgG4/7 on day 14 after vaccination (both p=0.033, r=0.886, **Supplementary Figure 6A**), and as a non-significant trend in the increases of CD4^+^TNF-α^+^ and IgG4/7 from day 0 to day 14 after vaccination (Δd0, p=0.058, r=0.829, **Supplementary Figure 6B**). Antibody and T cell responses to TT vaccination were not broadly predictive of each other in the horses used here.

## Discussion

The *in vivo* model of tetanus vaccination used in this study was suitable to analyze equine adaptive immune responses against a well-defined antigen. Antigen-reactive T cells were detected by CD154 induction as a new indicator along with cytokines, and antibodies confirmed the immune response as well-established markers. As expected, the horses had pre-existing immunity before vaccination mainly marked by TT-specific serum IgG3/5 and TT-reactive Th cells expressing TNF-α or IL-4. Nevertheless, vaccination induced an additional, mixed immune response with increased TT-specific serum antibodies of several isotypes, as previously described (39, 40). Nonetheless, this boosted response was again dominated by IgG3/5, which increased by a larger margin than TT-specific IgG1 or IgG4/7 by day 14 after vaccination, pointing to a bias toward a humoral response and indicative of Th2 cell help (1).

Increases in reactive T helper cells were detected in parallel to the antibody response, as elevated CD4^+^CD154^+^, and CD4^+^cytokine^+^ net frequencies in TT-re-stimulated PBMC after vaccination. This is in line with human Th cell activation indicated by CD154 and cytokine induction after re-stimulation with TT *in vitro* (15, 41). Equine T cell responses against tetanus have rarely been analyzed in detail. Proposing tetanus as a suitable model to study antigen-specific T cell responses in the horse Frayne and Stokes, 1995 detected proliferation of non-adherent cells from equine mandibular lymph nodes only, but not in PBMC (13), while McKelvie et al., 1998 demonstrated proliferation in PBMC (14). The phenotypes of the proliferating cells were not identified in these studies, but others demonstrated B cell stimulation (42). However, Hamza et al., 2013 demonstrated proliferation of Th cells in response to tetanus toxoid and confirmed regulatory T cell responses by inhibition of this proliferation (12). Further, comprehensive information on equine TT-specific Th cells and their dynamics has not been reported before. The detailed analysis has been limited by the lack of suitable indicators of specific T cell activation and of sensitive detection methods in light of the generally low frequency of antigen-specific T cells (8).

Similar to the application of CD154 as a marker of Th cell activation in mice (17, 19), humans (8, 41), and pigs (25, 26), we demonstrated its suitability for equine Th cells and identified specific cross-reactivity of the anti-human antibody 5C8 with equine CD154. The main stimulation effect of SEB or PMA combined with ionomycin on membrane-bound CD154 (38 kDa) detection in Western blots matches the use of cell lysates in this experiment. The stronger effect of SEB compared to PMA/ionomycin is however contrary to flow cytometry results, which yielded higher CD154^+^ frequencies with PMA/ionomycin than with SEB stimulation (see **Supplementary Figure 3**). The discrepancy may be due to higher viability after SEB compared to PMA/ionomycin stimulation and accordingly higher retention of membrane-bound CD154 for the detection in the lysates.

The largest proportion of CD154 expressing cells were CD4^+^ Th cells after re-stimulation. These also showed the most marked increase in TT-reactive net % after vaccination further supporting CD154 application to identify antigen-reactive Th cells in horses. Nevertheless, the consistent detection of CD4^-^CD154^+^ cells in equine PBMC is remarkable. CD4^-^CD154^+^ cells were a mixed population of several leukocyte subsets (see **Supplementary Figure 2**). This is in line with descriptions of CD154 expression on several leukocytes in humans and mice (17, 21). CD154 on non-T cells should be considered for the application of CD154 as an activation marker and warrants the combination with surface markers to specifically analyze T cells. Apart from T cells, we could identify some monocytes and B cells marked by anti-CD154, and a population negative for the surface markers available. These cells likely include platelet aggregates, which are usually contained in equine PBMC processed by standard methods and platelets are described as a major source of CD154 in other species (21, 43).

The accumulation of cytokine expressing cells in equine CD154^+^ Th cells further supports CD154 as an activation marker as described in other species (8, 17, 19, 25). However, the differences between TT re-stimulated CD154 induction and cytokine expression (in particular TNF-α, and IFN-γ) and the lack of stringent co-expression suggest that these markers of equine T cell activation are not simply interchangeable, but rather provide additional information on T cell reactivity. CD154 did not mark all equine cytokine expressing Th cells. This is in contrast to human PBMC analyses (15, 41). In PBMC from individuals who did not receive an additional booster vaccination before blood sampling, TT-reactive Th cells could be detected, and the vast majority of cytokine expressing CD4^+^ cells were CD154^+^ after re-stimulation with several antigens, including TT. Furthermore, human TT-reactive Th cells had a clear preferential Th1 signature expressing CD154, TNF-α, and IFN-γ, but hardly any IL-4 (41). In vaccination-boosted humans TT-reactive Th cells of several polarizations could be detected, but those with a Th2 cytokine signature were less abundant than Th1 cells in PBMC (44). In this study, the different Th cell expressed cytokines had distinct dynamics after vaccination, similar to our results in the present study with equine PBMC.

Similar to the results in humans, TNF-α and IFN-γ were detected in higher percentages of equine Th cells than IL-4 in our current study. Nevertheless, the course of net % increase over time differed between CD154 and the cytokines in putative Th1 cells (CD4^+^TNF-α^+^ and IFN-γ^+^), whereas for CD4^+^CD154^+^ and CD4^+^IL-4^+^ Th2 cells a similar time course was observed. Furthermore, the positive correlations of CD154 and IL-4 and negative correlations with IFN-γ indicate that the vaccination-induced increased frequencies of CD154 expressing TT-reactive Th cells may be associated with circulating Th2 cells (IL-4^+^) rather than Th1 cells (IFN-γ^+^). Compared to humans (15, 41), the equine immune response to tetanus vaccination appeared more type-2 biased here, as indicated by the IgG3/5 dominance of the serum antibodies (1).

The detection of several TT-specific IgG isotypes indicates T cell help for the class switching. A positive correlation of the circulating T helper cells and antibodies was detected for CD4^+^TNF-α^+^ T helper cells and IgG4/7 matching the previously reported association of Th1 responses with this isotype (1). Most other comparisons however, including those of TT-reactive CD4^+^CD154^+^ with serum Ig were independent (**Supplementary Figure 6**). The most abundant isotype, IgG3/5, was already detected before booster vaccination and independent from Th cell responses. As all horses had been vaccinated before, it is likely that the T cell help aiding the class switch to the dominating IgG3/5 had already occurred previously and the antigen alone was now sufficient to stimulate additional Ig secretion. Comparisons with more polarized responses favoring type 1 responses, such as viral infections (5, 7, 9) or type 2 responses, such as allergies (3, 11) are likely beneficial to analyze the association of equine T - and B cell responses in more detail.

## Conclusions

We demonstrated that tetanus toxoid vaccination is a well suitable model to analyze equine Th cell responses. Analyses of equine antigen-reactive T cells can be accomplished by the analysis of CD154 expression by the mAb 5C8 with confirmed cross-reactivity with the equine CD154 molecule in its cleaved (sCD154) and membrane-bound form. CD154 expression indicates T cell activation and marks activated Th cells enriched in cytokine producers. Nevertheless, it seems advisable to apply both, cytokine and CD154 analyses as complementary indicators of equine antigen-reactive T cells analyzed by flow cytometry. CD154 can be a useful new marker of equine T cell responses, which had not been well characterized in previous studies.

## Data Availability Statement

The original contributions presented in the study are included in the article/**Supplementary Material**. Further inquiries can be directed to the corresponding author.

## Ethics Statement

The animal study was reviewed and approved by Landesdirektion Sachsen.

## Author Contributions

CS designed the study, acquired funding for the analyses, acquired and processed samples and conducted experiments, drafted and finalized the manuscript. BF acquired the samples and conducted experiments. SL processed samples and conducted experiments. EM performed preliminary experiments and aided the study design. BW developed critical methods and provided antibodies for most analyses. GA aided the study design and funding acquisition, and participated in data interpretation and in writing the manuscript. All authors contributed to the article and approved the submitted version.

## Funding

CS and SL as well as materials and publication are funded by the German Research Foundation (DFG), Emmy-Noether-Programme, project number 431342499. The author(s) acknowledge support from the German Research Foundation (DFG) and Universität Leipzig within the program of Open Access Publishing.

## Conflict of Interest

The authors declare that the research was conducted in the absence of any commercial or financial relationships that could be construed as a potential conflict of interest.

## Publisher’s Note

All claims expressed in this article are solely those of the authors and do not necessarily represent those of their affiliated organizations, or those of the publisher, the editors and the reviewers. Any product that may be evaluated in this article, or claim that may be made by its manufacturer, is not guaranteed or endorsed by the publisher.

## Supplementary Material

Supplementary Table 1 Horses used.

Supplementary Figure 1 ELISA Standardization. Tetanus toxoid (TT) -specific serum antibodies were quantified by ELISA. Relative standards were used for quantification: (i) a pool serum from three horses with high antibody concentrations and (ii) Ig purified from the pool serum by Protein G, eluted in two steps at pH3, and at pH2. On TT-coated, blocked ELISA plates 2-fold serial dilutions of the pool serum and the purified Ig were applied and a serum from an unvaccinated horse (negative control) as well as a buffer control (blank) were included on each plate in addition to all serum samples. TT-specific antibodies were detected as pan-Ig and for three isotypes [IgG1, IgG3/5, IgG4/7] by biotinylated mAbs, followed by Streptavidin-HRP. After TMB incubation and stopping by phosphoric acid, ODs were quantified at 450 nm and blank-reduced by the buffer control. **(A)** Representative standard titration curves are displayed as blank-reduced OD vs. dilution (pool serum) or concentration (purified Ig). The horizontal dotted line represents the OD of the negative control serum (not shown for IgG4/7, below 0.125). **(B)** Medians and interquartile ranges of TT-specific Ig for the horses in this study are plotted over time normalized to pool serum (U/ml, right y-axes), or to purified Ig (µg Ig equivalent/ml, left Y-axes) as a standard. *days 0–14 n=7; day 28 n=5*

Supplementary Figure 2 Anti-human CD154 clone 5C8 detects CD154 in CD4^+^ and CD4^-^ equine PBMC. Whole PBMC, magnetically-sorted (MACS), CD4-enriched (CD4^+^), and CD4-depleted (CD4^-^) fractions were medium-incubated or stimulated *in vitro*. **(A)** Aliquots of PBMC and the sorted fractions were analyzed by flow cytometry before *in vitro* stimulation. After doublet exclusion and selection of live cells, CD4 staining was plotted against the side scatter (SSC) with percentages of CD4^+^ cells of all live singlets annotated. **(B, C)** Lysates of equine PBMC and MACS-sorted fractions were separated by SDS PAGE. Molecular weights (MW) in kDa are annotated as indicated by pre-stained MW markers included. **(B)** Proteins were blotted and visualized by StainFree technology (SF). **(C)** The Western blots were probed with anti-human CD154 conjugated with AF647 and detected as Cy5 fluorescence. CD154 bands are indicated by arrows. Note that the bands’ CD154/protein ratios were similar between the four samples analyzed, but Cy5 detection appears different due to uneven loading (compare B).

Supplementary Figure 3 Phenotypes of equine CD154^+^ PBMC. Equine PBMC (healthy horses, n=2) were used after isolation (*ex vivo*), incubated in medium or stimulated with SEB or PMA and ionomycin *in vitro* for 4h. PBMC were fixed and stained for flow cytometric analysis for CD154 (intracellular stain) and several combinations of the surface markers CD4, CD8, Ig, CD14, NKp46, and CD25 (see table). A representative example is shown. In all live PBMC **(A)**, CD154 was analyzed against each surface marker in quadrant gates **(B)**.

Supplementary Figure 4 Phenotypes of equine CD154^+^ CD4^-^CD8^-^ PBMC. Equine PBMC (healthy horses, n=2) were used after isolation (*ex vivo*), incubated in medium or stimulated with SEB or PMA and ionomycin *in vitro* for 4h. PBMC were fixed and stained for flow cytometric analysis for CD154 (intracellular stain) and the surface markers CD4, CD8, Ig, and CD14. A representative example is shown. **(A)** Singlets were gated (FSC-H vs. FSC-A) followed by live cells (exclusion of viability dye Fluor506-positive cells). **(B)** In all live PBMC, CD154^+^ were gated (polygon gates). Within these, CD4 and CD8 were analyzed (quadrant gates) and CD154^+^CD4^-^CD8^-^ cells were further analyzed for CD14 and Ig expression (quadrant gates).

Supplementary Figure 5 IL-4 expression in CD154 subpopulations of PBMC. On day 14 after vaccination PBMC (n=6) were medium-incubated or re-stimulated with tetanus toxoid (TT), the tetanus vaccine (Tet vax, ISCOM based), or SEB *in vitro* for 5h and analyzed by flow cytometry. Representative examples of one horse (H01) are depicted in pseudocolor plots with the dots enlarged for visibility. **(A)** Singlet-live-lymphocytes were gated and analyzed with quadrant gates for CD4 vs CD154, CD4 vs. IL-4, and CD154 vs IL-4. **(B)** In live lymphocytes CD4^+^ and CD4^-^ lymphocytes were gated (as the sum of 2 quadrants against CD8) and each fraction was further analyzed in quadrant gates for simultaneous expression of CD154 and IL-4.

Supplementary Figure 6 Tetanus toxoid-reactive T helper cell frequencies and TT-specific antibody concentrations were mainly independent. PBMC (n=6) were re-stimulated with tetanus toxoid (TT) *in vitro* compared to medium incubation and net % CD4^+^CD154^+^ and CD4^+^cytokine^+^ of live lymphocytes analyzed by flow cytometry. TT-specific serum antibody concentrations were quantified by ELISA for pan-Ig and the isotypes IgG1, IgG3/5 and IgG4/7. T cell activation markers are color-coded while Ig isotypes are indicated by the shapes of the symbols. **(A)** Net % of TT-reactive Th cells are plotted against serum antibody concentrations on day 0 (open symbols) and day 14 (filled symbols). * indicates Spearman correlation (p<0.05) of CD4^+^TNF-α^+^ and pan-Ig or IgG4/7 on day 14 (both comparisons p=0.033, r=0.886). All other parameters compared did not correlate. **(B)** Increases (Δd0) were calculated as the difference between day 14 and pre-vaccination for net %, or concentrations. Increases of TT-reactive Th cells are plotted against antibody concentration increases. CD4^+^TNF-α^+^ Δd0 and IgG4/7 Δd0 showed moderate correlation (p=0.058, r=0.829). All other parameters compared did not correlate.

## Abbreviations

CD, cluster of differentiation; Δd0, difference after vaccination – pre-vaccination; FCS, fetal calf serum; FSC, forward scatter; Ig, immunoglobulin; ISCOM, immune stimulating complexes; kDa, kilo Dalton; LD, live-dead; mAb, monoclonal antibody; MHC II, Major Histocompatibility Complex class II; MW, molecular weight; PAGE, polyacrylamide gel electrophoresis; PBMC, peripheral blood monocuclear cells; PBS, phosphate buffered saline; PMA, Phorbol-12-myristat-13-acetat; ns, not statistically significant; *r*, recombinant; RNA, ribonucleic acid; rt, room temperature; SEB, Staphylococcal enterotoxin B; SSC, side scatter; SDS, sodium dodecyl sulfate; Th, T helper; TT, tetanus toxoid.
