# Supplementary material for: CD154 Expression Indicates T Cell Activation Following Tetanus Toxoid Vaccination of Horses
Source: Front Immunol. 2022 Apr 13;13:805026. doi: 10.3389/fimmu.2022.805026 (PMC9043809; doi:10.3389/fimmu.2022.805026)
Supplement: Supplementary Table 1 — Horses used. [file Table_1.pdf]

Supplementary table 1 horses used

**ID**	**Age (years)**	**Breed**	**Last tetanus vaccination before this study (months)**
H01	15	Oldenburg horse	29
H02 #	16	Westphalian	40
H03	18	Mecklenburger	29
H04 #	18	Trakehner	40
H05	16	Oldenburg horse	33
H06	12	Hanoverian	15

unavailable for sampling on day 28 after vaccination
