# Supplementary figures and images for: CD154 Expression Indicates T Cell Activation Following Tetanus Toxoid Vaccination of Horses
Source: Front Immunol. 2022 Apr 13;13:805026. doi: 10.3389/fimmu.2022.805026 (PMC9043809; doi:10.3389/fimmu.2022.805026)

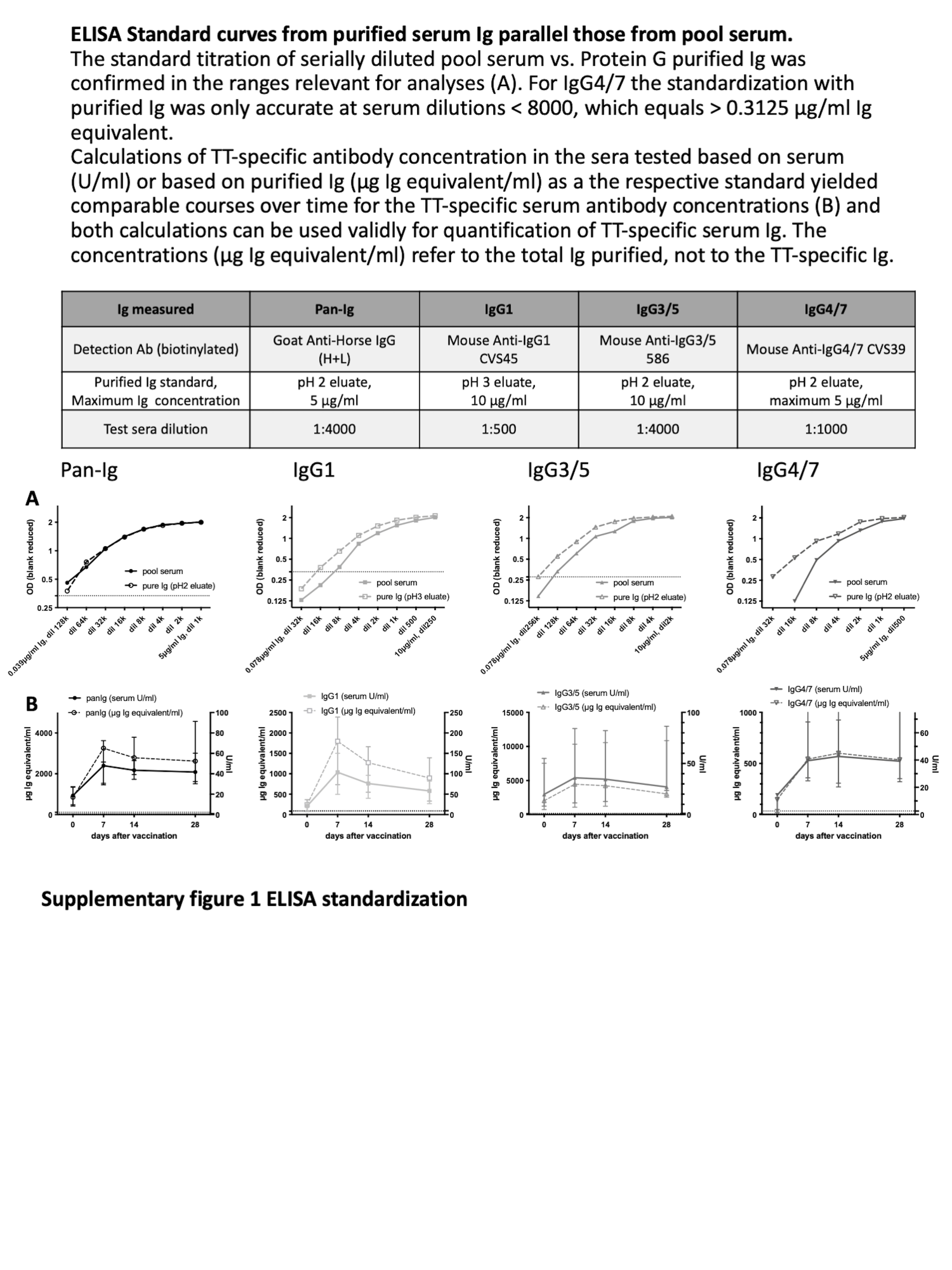

Supplement: Supplementary Figure 1 — ELISA Standardization. Tetanus toxoid (TT) -specific serum antibodies were quantified by ELISA. Relative standards were used for quantification: (i) a pool serum from three horses with high antibody concentrations and (ii) Ig purified from the pool serum by Protein G, eluted in two steps at pH3, and at pH2. On TT-coated, blocked ELISA plates 2-fold serial dilutions of the pool serum and the purified Ig were applied and a serum from an unvaccinated horse (negative control) as well as a buffer control (blank) were included on each plate in addition to all serum samples. TT-specific antibodies were detected as pan-Ig and for three isotypes [IgG1, IgG3/5, IgG4/7] by biotinylated mAbs, followed by Streptavidin-HRP. After TMB incubation and stopping by phosphoric acid, ODs were quantified at 450 nm and blank-reduced by the buffer control. (A) Representative standard titration curves are displayed as blank-reduced OD vs. dilution (pool serum) or concentration (purified Ig). The horizontal dotted line represents the OD of the negative control serum (not shown for IgG4/7, below 0.125). (B) Medians and interquartile ranges of TT-specific Ig for the horses in this study are plotted over time normalized to pool serum (U/ml, right y-axes), or to purified Ig (µg Ig equivalent/ml, left Y-axes) as a standard. days 0–14 n=7; day 28 n=5 [file Image_1.jpeg]

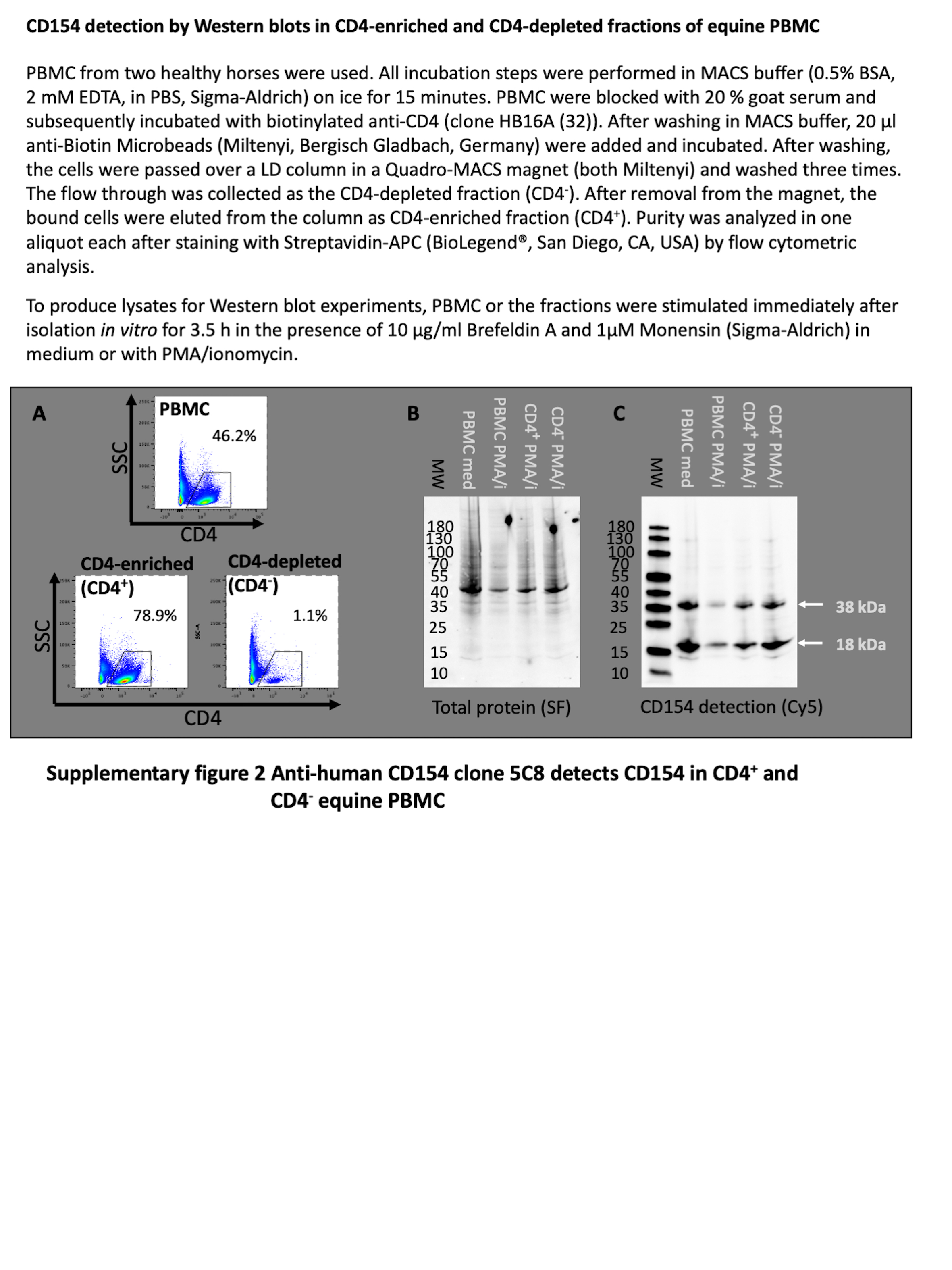

Supplement: Supplementary Figure 2 — Anti-human CD154 clone 5C8 detects CD154 in CD4+ and CD4- equine PBMC. Whole PBMC, magnetically-sorted (MACS), CD4-enriched (CD4+), and CD4-depleted (CD4-) fractions were medium-incubated or stimulated in vitro. (A) Aliquots of PBMC and the sorted fractions were analyzed by flow cytometry before in vitro stimulation. After doublet exclusion and selection of live cells, CD4 staining was plotted against the side scatter (SSC) with percentages of CD4+ cells of all live singlets annotated. (B, C) Lysates of equine PBMC and MACS-sorted fractions were separated by SDS PAGE. Molecular weights (MW) in kDa are annotated as indicated by pre-stained MW markers included. (B) Proteins were blotted and visualized by StainFree technology (SF). (C) The Western blots were probed with anti-human CD154 conjugated with AF647 and detected as Cy5 fluorescence. CD154 bands are indicated by arrows. Note that the bands’ CD154/protein ratios were similar between the four samples analyzed, but Cy5 detection appears different due to uneven loading (compare B). [file Image_2.jpeg]

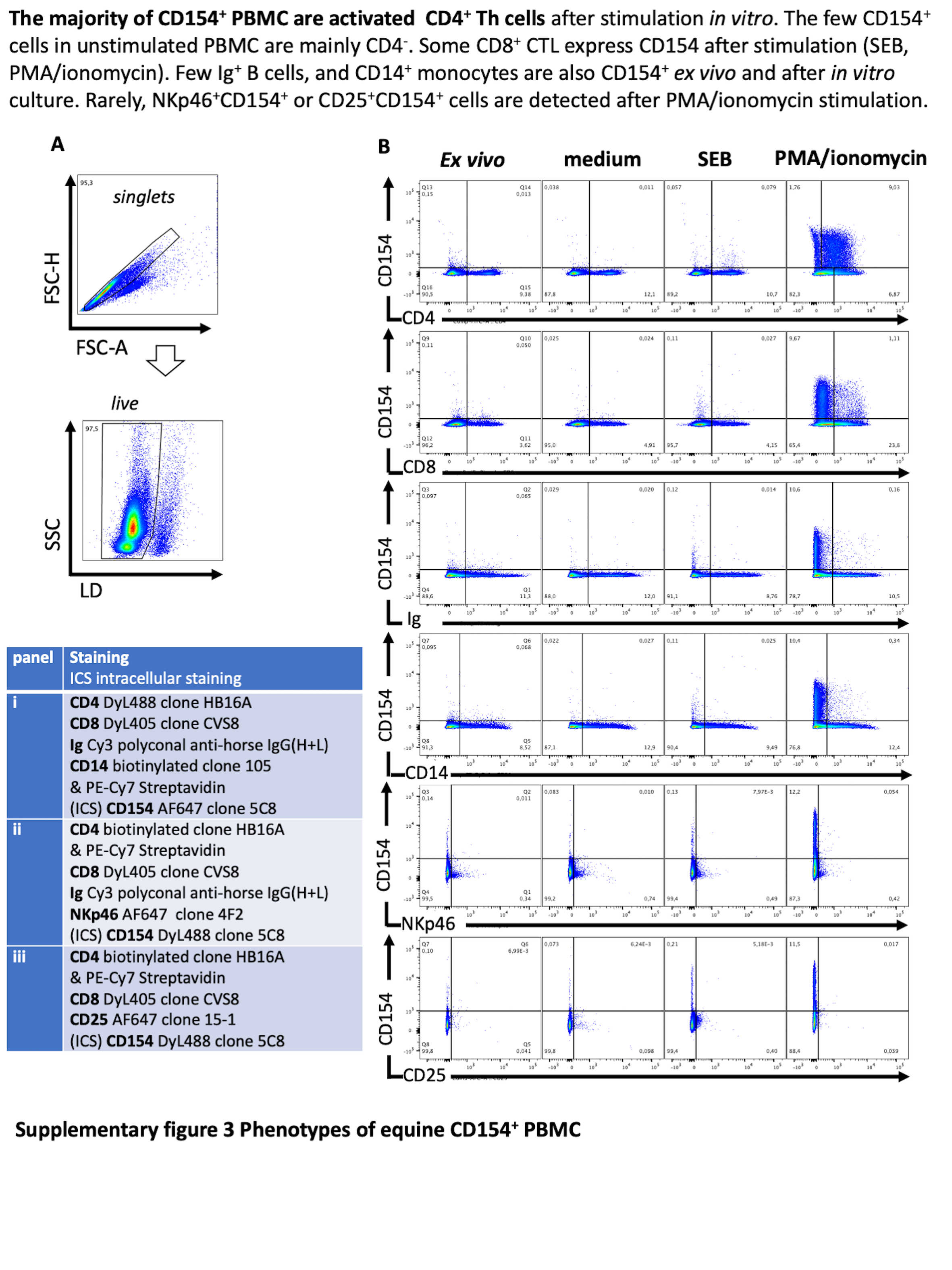

Supplement: Supplementary Figure 3 — Phenotypes of equine CD154+ PBMC. Equine PBMC (healthy horses, n=2) were used after isolation (ex vivo), incubated in medium or stimulated with SEB or PMA and ionomycin in vitro for 4h. PBMC were fixed and stained for flow cytometric analysis for CD154 (intracellular stain) and several combinations of the surface markers CD4, CD8, Ig, CD14, NKp46, and CD25 (see table). A representative example is shown. In all live PBMC (A), CD154 was analyzed against each surface marker in quadrant gates (B). [file Image_3.jpeg]

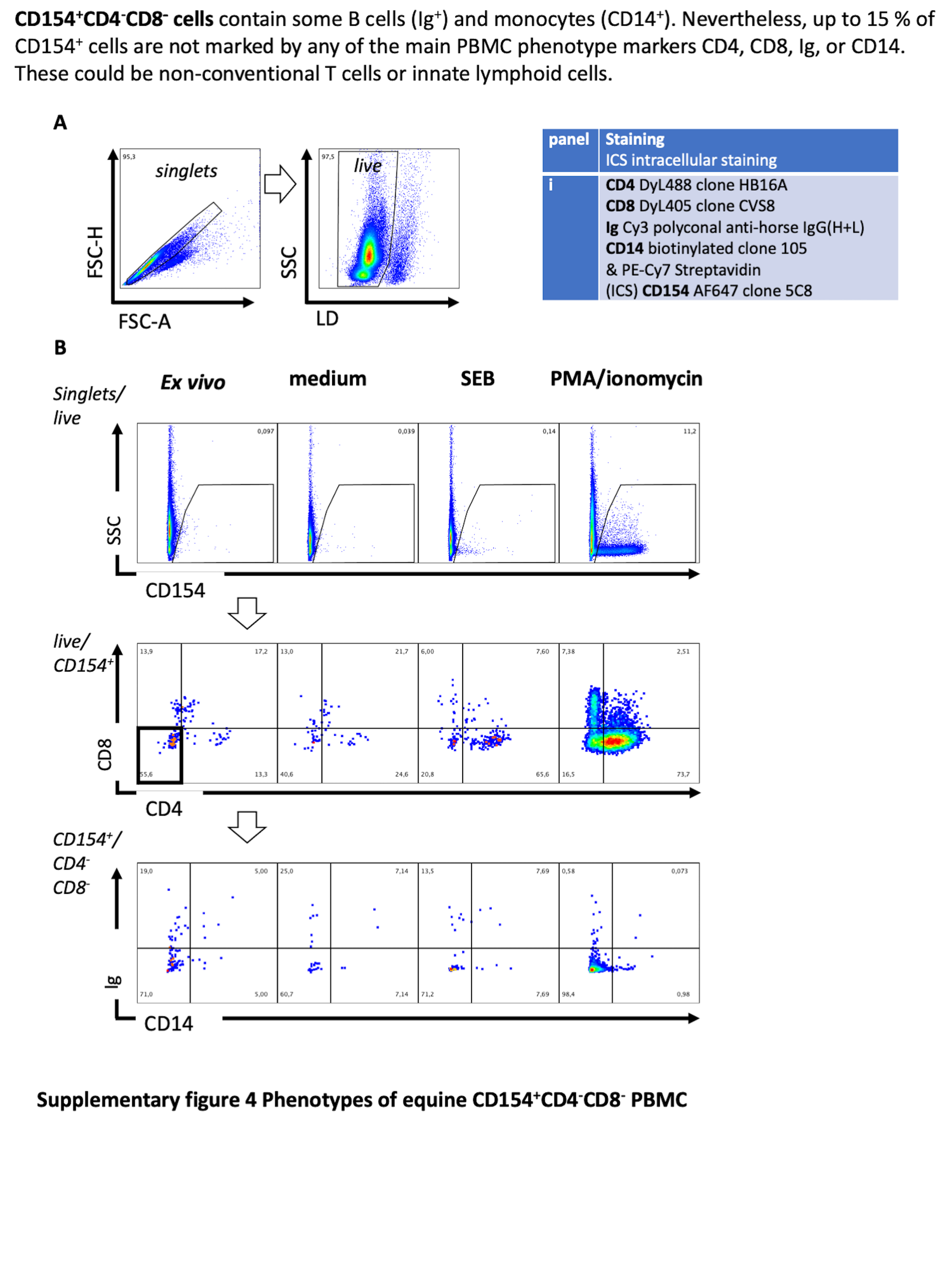

Supplement: Supplementary Figure 4 — Phenotypes of equine CD154+ CD4-CD8- PBMC. Equine PBMC (healthy horses, n=2) were used after isolation (ex vivo), incubated in medium or stimulated with SEB or PMA and ionomycin in vitro for 4h. PBMC were fixed and stained for flow cytometric analysis for CD154 (intracellular stain) and the surface markers CD4, CD8, Ig, and CD14. A representative example is shown. (A) Singlets were gated (FSC-H vs. FSC-A) followed by live cells (exclusion of viability dye Fluor506-positive cells). (B) In all live PBMC, CD154+ were gated (polygon gates). Within these, CD4 and CD8 were analyzed (quadrant gates) and CD154+CD4-CD8- cells were further analyzed for CD14 and Ig expression (quadrant gates). [file Image_4.jpeg]

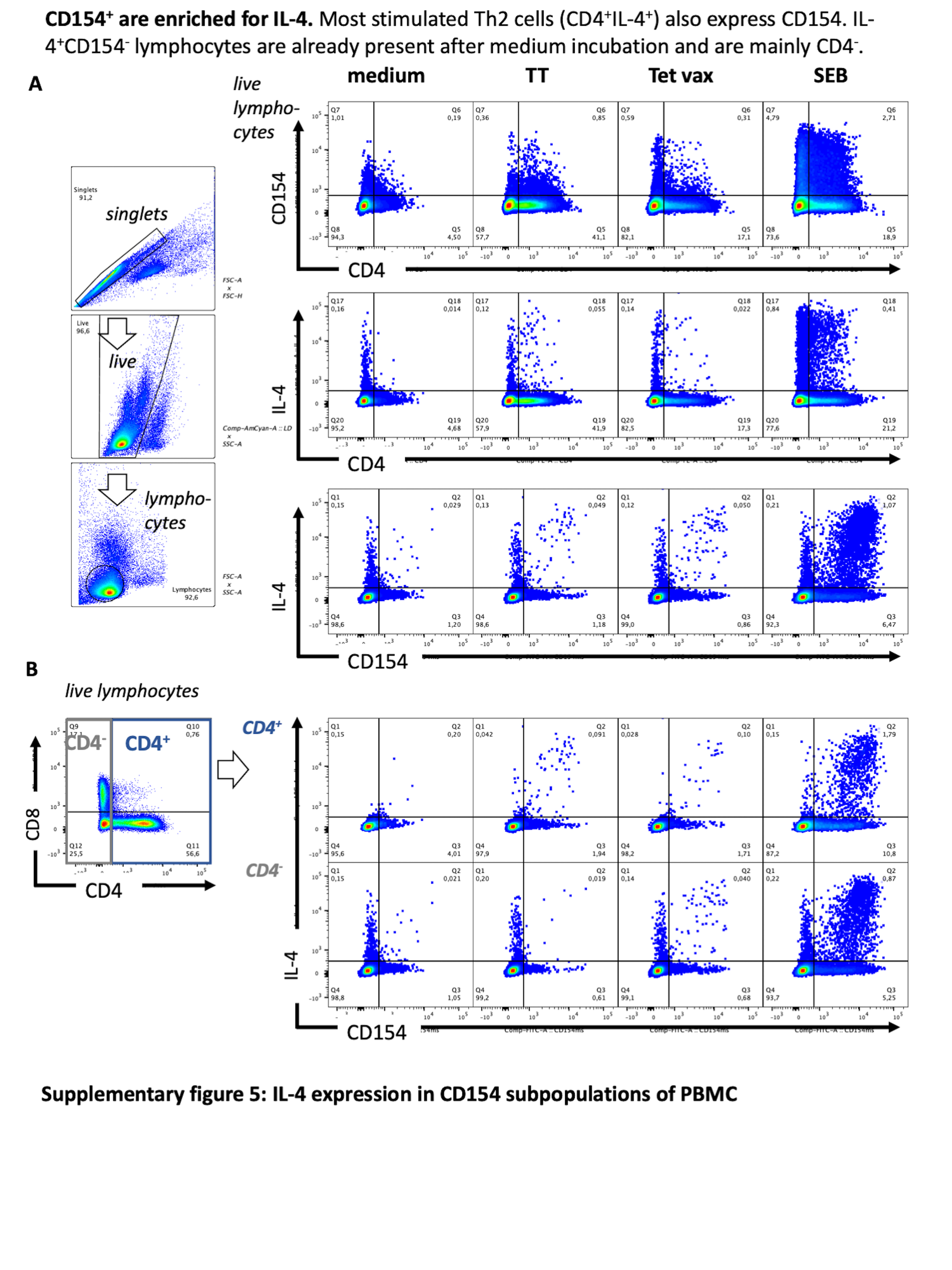

Supplement: Supplementary Figure 5 — IL-4 expression in CD154 subpopulations of PBMC. On day 14 after vaccination PBMC (n=6) were medium-incubated or re-stimulated with tetanus toxoid (TT), the tetanus vaccine (Tet vax, ISCOM based), or SEB in vitro for 5h and analyzed by flow cytometry. Representative examples of one horse (H01) are depicted in pseudocolor plots with the dots enlarged for visibility. (A) Singlet-live-lymphocytes were gated and analyzed with quadrant gates for CD4 vs CD154, CD4 vs. IL-4, and CD154 vs IL-4. (B) In live lymphocytes CD4+ and CD4- lymphocytes were gated (as the sum of 2 quadrants against CD8) and each fraction was further analyzed in quadrant gates for simultaneous expression of CD154 and IL-4. [file Image_5.jpeg]

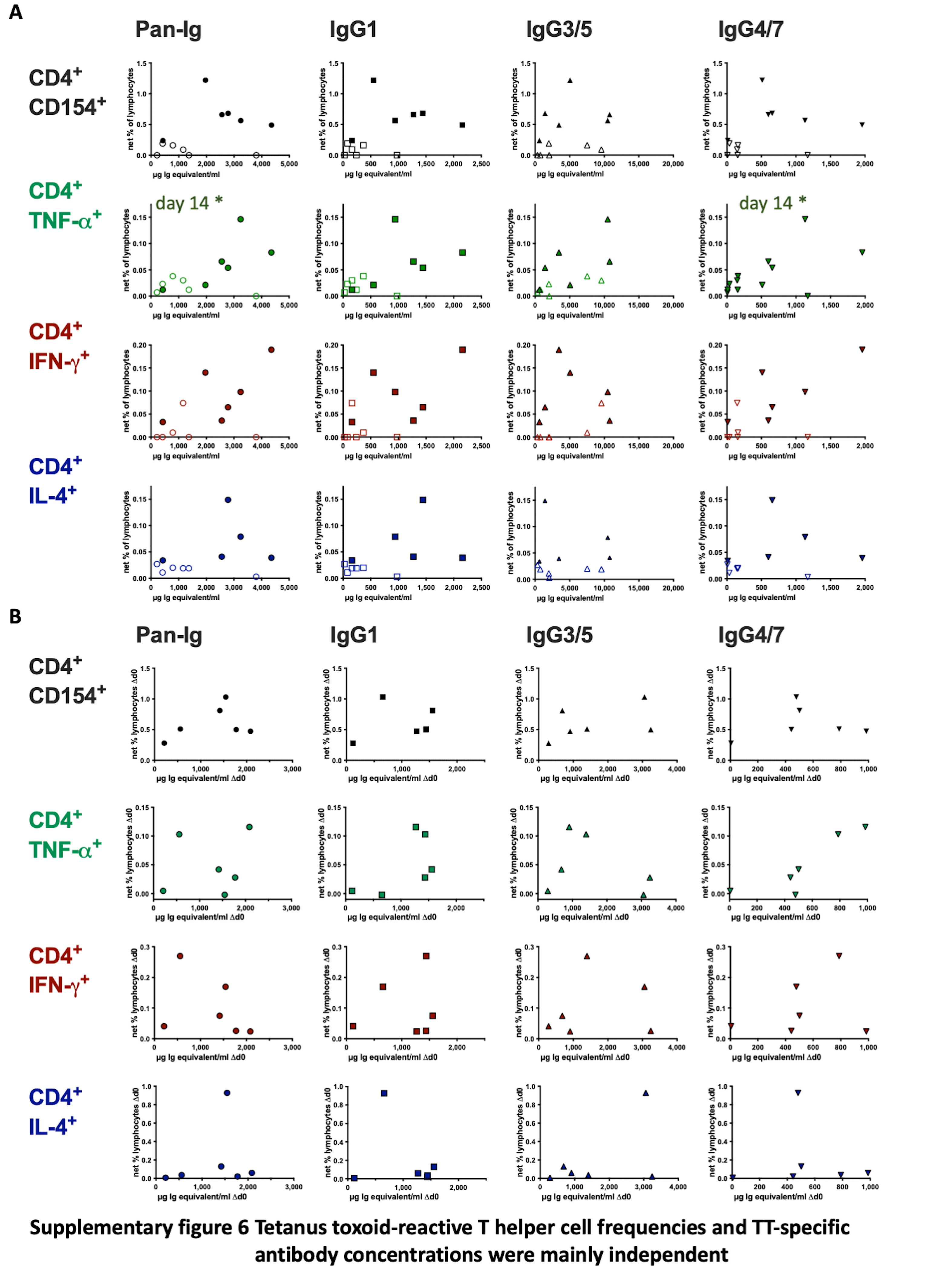

Supplement: Supplementary Figure 6 — Tetanus toxoid-reactive T helper cell frequencies and TT-specific antibody concentrations were mainly independent. PBMC (n=6) were re-stimulated with tetanus toxoid (TT) in vitro compared to medium incubation and net % CD4+CD154+ and CD4+cytokine+ of live lymphocytes analyzed by flow cytometry. TT-specific serum antibody concentrations were quantified by ELISA for pan-Ig and the isotypes IgG1, IgG3/5 and IgG4/7. T cell activation markers are color-coded while Ig isotypes are indicated by the shapes of the symbols. (A) Net % of TT-reactive Th cells are plotted against serum antibody concentrations on day 0 (open symbols) and day 14 (filled symbols). * indicates Spearman correlation (p<0.05) of CD4+TNF-α+ and pan-Ig or IgG4/7 on day 14 (both comparisons p=0.033, r=0.886). All other parameters compared did not correlate. (B) Increases (Δd0) were calculated as the difference between day 14 and pre-vaccination for net %, or concentrations. Increases of TT-reactive Th cells are plotted against antibody concentration increases. CD4+TNF-α+ Δd0 and IgG4/7 Δd0 showed moderate correlation (p=0.058, r=0.829). All other parameters compared did not correlate. [file Image_6.jpeg]
